# Identification of EEG features during status epilepticus for prediction of emergent epilepsy phenotype in the mouse intra-amygdala kainic acid model using supervised learning

**DOI:** 10.1016/j.ibneur.2026.04.013

**Published:** 2026-05-02

**Authors:** Syed Muhammad Raza Abidi, Omar Mamad, Jordan Higgins, David C. Henshall, Gabriel-Miro Muntean

**Affiliations:** a Performance Engineering Lab (PEL), School of Electronic Engineering, Insight Research Ireland Centre, Dublin City University, Dublin, Ireland; b Department of Physiology and Medical Physics and FutureNeuro Research Ireland Centre, RCSI University of Medicine and Health Sciences, Dublin, Ireland

**Keywords:** Seizure burden severity prediction, EEG biomarkers, Baseline recordings, Kainic acid mouse model, Machine learning

## Abstract

Preclinical animal models are essential for investigating epilepsy mechanisms and evaluating novel therapies. In rodents, epilepsy can be induced by status epilepticus, leading to later spontaneous recurrent seizures (SRSs). However, inter-animal variability in seizure burden can limit suitability for drug studies. Here, we investigated whether early electroencephalography (EEG) recordings acquired during status epilepticus (∼40 min) can predict the later burden of spontaneous seizures in the intra-amygdala kainic acid (IAKA) model in mice. Spectral and statistical EEG features were extracted from IAKA model mice (n = 19) and used to train supervised classifiers, including Random Forest, Support Vector Machine (SVM), and Logistic Regression. Performance was evaluated using Leave-One-Out and 5-fold-stratified cross-validation. Feature robustness was enhanced through an intersection-based strategy combining ANOVA, Mutual Information, Random Forest importance, and SHAP analysis, together with engineered change-based (∆) features derived as post-KA minus baseline activity. Among the evaluated models, SVM achieved the strongest internal performance (weighted F1-score: 0.74), demonstrating that early EEG dynamics during status epilepticus encode prognostic information related to later SRSs burden. Importantly, the model reliably identified animals within the intermediate (normal) seizure-burden group that showed an average of two-week seizure count of 53 ± 11, relative to low (18 ± 11) and high (85 ± 43) groups. While generalization to an independent validation cohort (n = 11) was limited, this likely reflects biological heterogeneity and duration-dependent EEG variability. Overall, these findings highlight the relevance of early electrographic activity in shaping seizure-burden outcomes and provide a foundation for future longitudinal prognostic studies.

## 1 Introduction

Epilepsy is a prevalent neurological disorder characterized by recurrent, unprovoked seizures and associated with cognitive decline, morbidity, and reduced quality of life. Electroencephalography (EEG) remains the gold-standard tool for assessing brain electrical activity and is routinely used in both clinical and preclinical settings to monitor seizure onset, interictal abnormalities, and treatment response. EEG biomarkers such as high frequency oscillations, shifts in spectral power within delta and theta bands, or changes in waveform symmetry have been shown to correlate with disease severity and prognosis in both humans and rodent models (Kanai et al., 2019, Song et al., 2024, Zijlmans et al., 2012).

Rodent models, including those induced by intra-amygdala kainic acid (IAKA), are widely used to replicate temporal lobe epilepsy (TLE) and can reproduce key pathophysiology within the brain and co-morbid features. After KA injection, an episode of status epilepticus (continuous seizures) develops which later abates and is followed by the emergence, within a few days, of spontaneous recurrent seizures (SRSs). Once epilepsy is established, mice in the IAKA model typically display 5–10 SRS per day (Mamad et al., 2023, Mouri et al., 2008, Reschke et al., 2021).

EEG analysis offers a window into brain dynamics during seizure induction, yet few studies have explored whether baseline or early post-KA EEG features can predict long term epilepsy severity outcomes (Puttachary et al., 2015, Sharma et al., 2018, White et al., 2010). After status epilepticus in the IAKA model, most animals develop a typical (normal) frequency of SRS. However, a number of individual mice will develop either too low or too high rates of SRS which make them unsuitable for testing experimental therapies or because of morbidity, respectively. Predicting which mice will develop too high or too low rates of SRS would reduce resource use and enable humane endpoints in preclinical trials. It is uncertain, however, if early post-KA recordings or quantitative EEG feature changes (e.g., post minus baseline differences) relate to subsequent spontaneous seizure burden phenotypes in this model.

Recent advances in machine learning (ML) offer opportunities to automate EEG signal analysis and apply this to seizure classification and prediction models in both clinical and preclinical epilepsy research (Acharya et al., 2012, Edoho et al., 2025, Sheikh et al., 2024). Notably, recent work has demonstrated that machine-learning models trained on preclinical EEG recordings made during the status epilepticus period can classify emergent phenotypes into normal vs outlier groups with moderate accuracy using both feature-based and transfer-learning strategies (Edoho et al., 2025).

Building on these advances, the present study aimed to identify early EEG features most predictive of subsequent spontaneous seizure burden phenotypes in the IAKA model, using supervised learning and systematic feature-selection strategies. Here, we leverage a supervised ML approach trained on labelled mouse EEG data to predict spontaneous seizure burden severity across three categories—*Low, Normal,* and *High*. Using per-mouse, recording-level features, we integrate both post-KA features and change-based (∆) post-KA minus baseline features that capture neural alterations following KA administration. A range of spectral, statistical, and Hjorth-based EEG features were extracted (Hjorth, 1975), and feature selection was guided by the intersection of ANOVA, Mutual Information, Random Forest, and SHAP importance ranking technique.

The resulting compact feature set was used to train and evaluate multiple classifiers under 5-fold StratifiedKFold cross-validation. To ensure transparency and biological interpretability, Explainable AI (XAI) methods—including Shapley Additive explanations (SHAP) and feature contribution visualization—were applied to quantify each feature’s role in predicting severity. This interpretability layer revealed distinct EEG patterns associated with low, normal, and high SRS rate phenotypes, particularly highlighting how post-KA mobility, theta activity, and waveform asymmetry collectively encode the severity spectrum. Together, the findings indicate that ML models can use early EEG recordings during status epilepticus in mice to predict subsequent spontaneous seizure burden phenotypes with meaningful accuracy, providing potential utility for stratifying animals in preclinical epilepsy research.

## 2 Materials & methods

### 2.1 Dataset and recordings

Mouse studies were carried out in accordance with the European Communities Council Directive (2010/63/EU). Procedures were approved by the Research Ethics Committee (REC 1587) of the RCSI University of Medicine and Health Sciences, under license from the Ireland Health Products Regulatory Authority (AE19127/P057). Animals were housed on a 12 h light-dark cycle under controlled conditions (temperature: 20°C–25°C; humidity: 40%–60%). Food and water were available ad libitum (Edoho et al., 2025). Animal studies were part of ongoing research into the causes and treatment of experimental epilepsy and were not specifically generated for this study.

Epilepsy was induced using the IAKA model technique. Briefly, male C57BL/6JOlaHsd mice (weight: 28–30 g; age 10 weeks) were anaesthetised with isoflurane (5% induction, 2% maintenance) and placed in a mouse-adapted stereotaxic frame. Next, four partial craniotomies were performed for affixing surface EEG. An EEG transmitter unit (model HDX-02; Data Systems International (DSI), MN, USA) was implanted under the skin in a subcutaneous pocket along the dorsal flank of the mouse. The main recording electrodes were placed bi-laterally, visually guided to lie above dorsal hippocampus; approximately −2.1 mm posterior, + /- 1.2 mm lateral from the Bregma, while avoiding the amygdala targeting cannula placement site. Two reference electrodes were placed anterior to these sites, also by visual guidance alone, approximately + 2.7 mm anterior and + /- 1.5 lateral on each side. After 48 h recovery, all animals received an intra-amygdala microinjection of KA (0.3 µg in 0.2 µL volume). Status epilepticus developed and was recorded using the surface EEG. After 40 min, all mice received an intraperitoneal (IP) injection of lorazepam (8 mg/kg; Pfizer) in order to reduce morbidity and mortality. Mice were returned to their home cages and stayed under climate-controlled conditions and video monitoring combined with telemetry. EEG was recorded from individually housed, freely moving mice for 24 h/day up to 5 weeks after KA injection (Mamad et al., 2023, Mouri et al., 2008, Reschke et al., 2021).

**Fig. 1:**
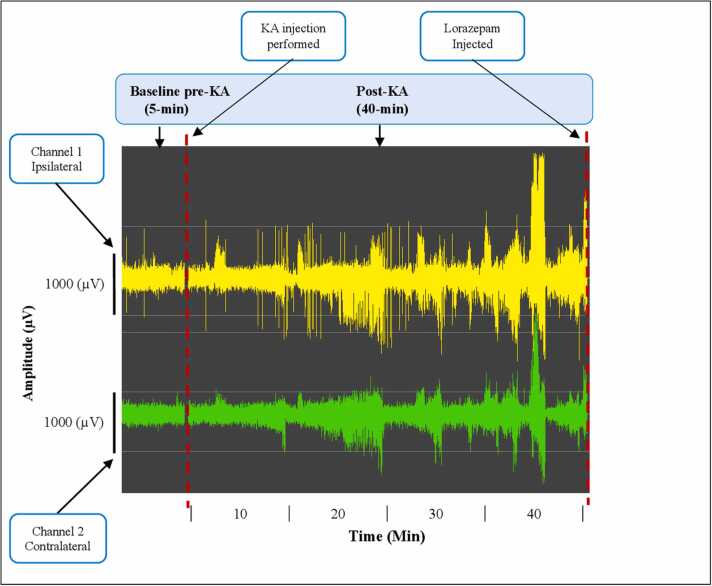
Status epilepticus after IAKA recorded in mice using two-channel telemetry EEG. Representative EEG trace from a mouse in the IAKA model. Trace shows (left) baseline EEG followed by the period after KA injection and the later (right/end) interruption of signal at the time when the mouse is removed from the recording station to administer lorazepam.

Continuous temperature and EEG data were collected from the implanted EEG devices (Ponemah v6.30 software, DSI). Files were exported for analysis using LabChart 8 Reader (ADInstruments, Oxford, UK). SRS events were defined as high amplitude (>2x baseline), high frequency (>5 Hz) and containing poly-spike discharges persisting for > 5 s. Parameters of interest including seizure duration and EEG power and frequency were extracted using this software. The number of spontaneous seizures were counted, and the epilepsy phenotype was assigned based on SRS burden during the 13 days post-KA using the mean SRS number/day ± 2 SEM from days 7–13 as upper and lower cut-offs for the SRS high (H) and SRS low (L) groups. For an individual mouse to be classified as high (H) or low (L) they had to exhibit an SRS number greater than mean + 2 SEM or less than mean – 2 SEM on a minimum of four days from days 7–13 post-KA, respectively. If these cut-offs were not met mice were classified as SRS normal (N). Any mice that died (e.g. SUDEPs) were also classified as SRS high (H). The EEG data used were therefore: (a) a baseline EEG recording of approximately 5 min collected before KA administration; and (b) a post-KA recording of approximately 40 min collected following KA injection (and prior to lorazepam administration). In total, EEG recordings from 30 mice were analyzed in this study. A primary cohort of 19 mice was used for model development, including training and internal validation, while an independent cohort of recordings from 11 mice was used exclusively for external validation of model performance. An example of an acute EEG recording before and after KA are shown in Fig. 1.

### 2.2 Preprocessing

**Fig. 2:**
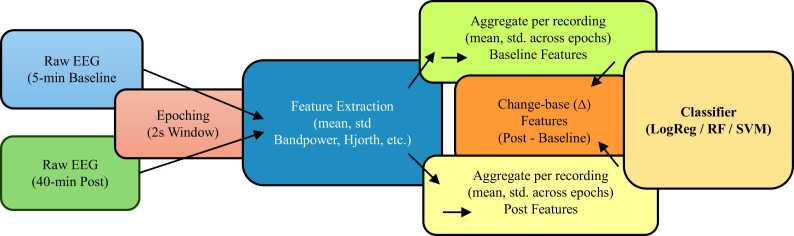
Mice-EEG feature extraction and classification workflow. Schematic shows the complete processing pipeline used for spontaneous seizure burden severity prediction from mice EEG recordings. Raw EEG signals recorded during baseline (5 min) and post-KA status epilepticus (40 min) were segmented into non-overlapping 2-second epochs. Time-domain, frequency-domain, and non-linear features (e.g., bandpower, Hjorth parameters) were extracted at the epoch level and subsequently aggregated per recording using summary statistics (mean and standard deviation). Baseline and post-KA features were then combined to derive change-based (∆) features representing post-KA minus baseline differences. The resulting feature sets were used to train supervised classifiers, including Logistic Regression, Random Forest, and Support Vector Machine models.

Several studies have used the status epilepticus period to predict the emergent phenotype (normal vs outlier) with feature-based and transfer learning approaches (Edoho et al., 2025, Peng et al., 2020); we adopt a complementary aim by focusing on change-based (∆) and multiclass severity. In this study the collected mice-EEG data have sampling frequency: 500 Hz, DC offset removal and bandpass filtering as needed (e.g., 0.5–80 Hz). Two hippocampal channels (Channel 1 ipsilateral and Channel 2 contralateral) were used for all analyses. Overall workflow pipeline is shown in Fig. 2.

### 2.3 Epoching and feature estimation

**Epoching:** Continuous recordings were segmented into consecutive non-overlapping epochs of 2 s, a standard practice in EEG analysis for spectral and statistical feature extraction (Gramfort et al., 2014). This ensured consistent temporal resolution for feature extraction. For baseline 5-min, this yields ∼155 (for 2 s) epochs per recording.

**Feature extraction (time & time–frequency):** For each epoch and each channel we computed spectral band powers and several time-domain and nonlinear features. The main feature set includes, per epoch and per channel are shown in Table 1. Features were computed separately for baseline, post-KA, and their change-based (∆) to capture dynamic signal shifts indicative of excitotoxic response.

**Table 1:** EEG Feature Extraction.

Time-Domain Features	Frequency-Domain Features
▪ Mean, Standard Deviation (Std.), Variance (Var), Root Mean Square (RMS) ▪ Skewness, Kurtosis ▪ Zero-crossing Rate (ZCR) ▪ Hjorth Parameters (Activity, Mobility, and Complexity)	▪ Delta (0.5, 4) Hz ▪ Theta (4, 8) Hz ▪ Alpha (8, 12) Hz ▪ Beta (12, 30) Hz ▪ Gamma (30, 80) Hz ▪ Relative band powers: rel_delta, rel_theta, rel_alpha, rel_beta, rel_gamma

**Feature matrix:** The epochs were then stored in a feature matrix $\left(\mathrm{rows}=\mathrm{epochs},\mathrm{columns}=\mathrm{features}\right)$. The shape of epochs per mouse for baseline pre-KA is $\left(155\mathrm{epochs}\;x40\mathrm{features}\right)$ 20 features per channel x 2 channels.

### 2.4 Recording-level aggregation

To obtain one feature vector per recording (mouse) for classification, epoch-level features were aggregated per recording by computing summary statistics (mean and standard deviation) for each feature across all epochs belonging to that recording. This yields a recording-level feature matrix with rows = recordings (mice) and columns = aggregated features (e.g., base_ch1_alpha_mean, base_ch1_alpha_std, etc.). Aggregation reduces within-subject correlation and produces one sample per subject so that model evaluation reflects subject-wise generalization.

Our rationale for aggregating EEG features at the mouse level using summary statistics (mean and standard deviation) was to ensure subject-level independence during model evaluation and to prevent epoch-level data leakage, whereby epochs from the same animal could otherwise appear in both training and testing sets.

**EEG frequency bands and biological meaning:** EEG signal usually can be divided in frequency bands, and every band has its own functional/clinical relevance.

o Delta (0.5–4 Hz): Shows it plays role in deep sleep, slow-wave activity, severity markers of epilepsy (Bernardi et al., 2019).
o Theta (4–8 Hz): Cortical theta rhythms playing a role in memory encoding, drowsiness, abnormal activity in epilepsy (Jarovi et al., 2018).
o Alpha (8–13 Hz): Playing a role in visual processing, relaxed wakefulness, visual cortex rhythms (van Kerkoerle et al., 2014).
o Beta (13–30 Hz): Motor processing & control, active thinking, abnormal increase in epilepsy (hyperexcitability) (Pierrieau et al., 2025).
o Gamma (30–80 Hz): Essentially gamma is not specific but is a metric of binding/computation of information processing, cognitive binding, visual processing, and seizure signatures (Fernandez-Ruiz et al., 2023).

*Power spectral density (PSD) calculation:*

$$\mathrm{PSD}\left(f\right)=\mathrm{Power}\;\mathrm{at}\;\mathrm{frequency}\;f$$

*Absolute band power:*

In absolute band power, we integrate PSDs of every band. For instance:

$$P_{\mathrm{alpha}}=\;\sum_{f=8}^{13}\mathrm{PSD}(f)$$

Same for $P_{\mathrm{delta}},\;P_{\mathrm{theta}},\;P_{\mathrm{beta}},\;\ldots$ calculation.

*Relative band power:*

Absolute powers vary mouse-to-mouse, electrode-to-electrode, or recording condition etc., so, we normalize the powers

$$\mathrm{rel}\_\mathrm{alpha}=\;\frac{P_{\mathrm{alpha}}}{P_{\mathrm{total}}}$$

where:

$$P_{\mathrm{total}}=\;P_{\mathrm{delta}}+\;P_{\mathrm{theta}}+\;P_{\mathrm{alpha}}+\;P_{\mathrm{beta}}+\;P_{\mathrm{gamma}}$$

### 2.5 Feature selection

Feature selection was performed to identify the most discriminative EEG biomarkers that contribute to predicting spontaneous seizure burden severity (Low, Normal, High). The process aimed to reduce redundancy, enhance model generalization, and improve interpretability of the final supervised model. EEG signals were segmented into 2 s epochs and transformed into the frequency domain using the Fast Fourier Transform (FFT). With a sampling frequency of 1000 Hz, the FFT size was 2000 samples, yielding a frequency resolution of 0.5 Hz. Only the one-sided spectrum was used for feature extraction. Initially, a Leave-One-Out Cross-Validation (LOOCV) strategy (Stone, 1974) was employed to evaluate individual feature importance and ensure stability across subjects. However, given the small sample size and potential sensitivity of LOOCV to inter-animal variability, the approach was refined to use 5-fold Stratified Cross-Validation (StratifiedKFold) (Kohavi, 1995), which provided more balanced partitions and yielded significantly more consistent results across folds.

#### 2.5.1 Hybrid multi-criterion selection approach

To robustly capture both statistical relevance and predictive contribution, four complementary feature ranking techniques were integrated:

**ANOVA F-test (univariate filter method):** Evaluates the variance ratio between and within severity classes for each feature, selecting those with statistically significant mean differences across Low, Normal, and High groups (Guyon and De, 2003).

**Mutual information (MI):** Quantifies the nonlinear dependency between each EEG feature and the severity labels, identifying features with the strongest information gain regarding class boundaries (Vergara and Estévez, 2014).

**Random forest (RF) feature importance:** Provides model-based importance scores reflecting how frequently and effectively each feature reduces Gini impurity during tree splits, thus highlighting discriminative features in a multivariate, nonlinear space (Breiman, 2001).

**Shapley additive explanations (SHAP) value ranking:** Applied after training ensemble models, SHAP measures the marginal contribution of each feature to the model’s prediction, offering a unified and explainable metric of global importance (Lundberg et al., 2017).

The intersection of top-ranked features from all four techniques (ANOVA, MI, RF, and SHAP) was taken to ensure that only consistently informative features were retained across statistical, information-theoretic, and model-based criteria. This intersection strategy yielded a compact and interpretable subset of key EEG features, comprising both change-based (∆) and post-KA-only metrics.

**Outcome and rationale:** This hybrid feature selection process achieved a strong balance between interpretability and predictive robustness. Compared to LOOCV-based selection, the StratifiedKFold-based hybrid intersection not only improved cross-validation stability but also enhanced classifier performance.

### 2.6 Classifiers and evaluation

**Classifiers:** Logistic Regression, Random Forest (200 trees), and SVM (RBF kernel). Hyperparameters were tuned using cross-validation where indicated.

**Cross-validation and test strategy:** To avoid data leakage we used subject-wise splits. For final evaluation we used Leave-One-Mouse-Out (LOMO) at subject level (as specified per experiment). LOOCV is preferred for small N (N = 19) to measure generalization to unseen mice. Then we used the StratifiedKFold CV for optimal best results.

**Metrics:** Primary metric: macro-averaged F1 score (equal weight to each class). We also report accuracy, class-wise precision and recall, and weighted averages. Permutation testing (n = 100) was used to assess whether performance exceeds chance.

### 2.7 Independent validation dataset

To assess the generalizability of the trained classifiers, an independent validation dataset was assembled comprising EEG recordings from an additional cohort of (N = 11) mice. These EEGs were acquired separately from the primary training dataset (N = 19). These animals were of the same strain as those used in the primary cohort, and status epilepticus was induced using the same IAKA protocol under identical experimental conditions. All data were processed using the same preprocessing, epoching, and feature extraction pipeline described above. Importantly, the validation dataset was not used at any stage of model training, feature selection, feature aggregation, or hyperparameter optimization. Model predictions on this dataset therefore represent a fully unseen evaluation intended to assess external performance under realistic experimental variability.

### 2.8 Implementation details

We used code implemented in Python 3, on Jupyter Notebook version (6.5.4) (pandas, scikit-learn, SHAP, matplotlib) (Kluyver et al., 2016, Pedregosa et al., 2011), and Python-MNE version (1.6.0) (Gramfort et al., 2013). Random seeds were fixed for reproducibility.

## 3 Results

### 3.1 Overview of experimental design and data processing

The present study used EEG recordings at the time of induction of status epilepticus by IAKA to predict the emergent spontaneous seizure burden phenotypes. A total of 19 mice were included in the primary training cohort, while an additional independent cohort of 11 mice was used for validation during status epilepticus for which seizure count means and standard deviations (SDs) for the two-week period shown in Table 2 of each group classified as low, normal, and high.

**Table 2:** Seizure count means and standard deviations for two-week period.

Group	Mean	Standard Deviation
Low	18.3	11.03
Normal	53.1	10.73
High	85.0	43.14

Notably, the objective of the study was to identify mice that will develop the normal seizure phenotype with as much accuracy as possible based on the first seizure burst after IAKA up to when lorazepam was injected as shown in Fig. 1. EEG recordings were collected in a European Data Format (.edf), then the pre-processed signals were segmented into two-second non-overlapping epochs. Epoching is the process of segmenting EEG signals into time intervals, and the signal within each time interval is known as an epoch. Features were extracted from the time and time-frequency domains. Each epoch of the pre-processed signals was used to estimate the characteristics from both domains.

**Fig. 3:**
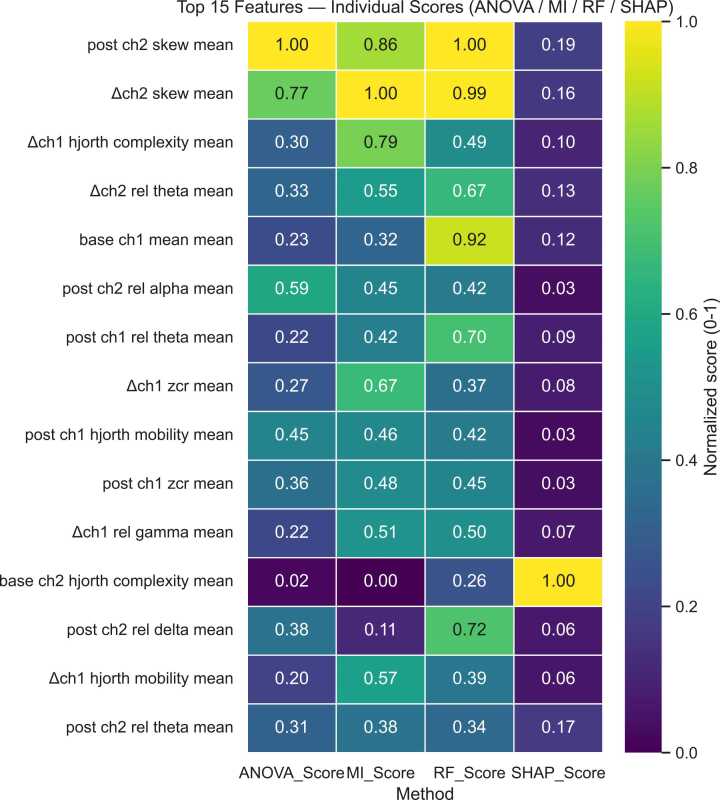
Heatmap illustrates the individual importance scores of the top 15 EEG features ranked independently using ANOVA, Mutual Information (MI), Random Forest (RF), and SHAP-based feature selection methods. Each column represents a feature selection technique, while colour intensity indicates the relative contribution of each feature to severity classification, with lighter shades reflecting higher importance.

We conducted experiments in two phases. In phase – 1, we evaluated model performance using top-15 features extracted from 4 techniques (i.e., ANOVA, MI, RF, and SHAP) shown in Fig. 3 and applied classification using LOOCV on recording-level and classifier comparison report shown in Table 3. It systematically evaluates the severity using individual feature selection technique using three classifiers (Random Forest, Logistic Regression, and SVM). The classifier used LOOCV ML technique. Random Forest and SVM achieved (F1 = 0.67 and F1 = 0.63) respectively using the MI selection technique, and Logistic Regression achieved (F1 = 0.68) using RF.

**Table 3:** Recording-level Classifier Comparison.

	**ANOVA** **Selected Features**				**MI** **Selected Features**				**RF** **Selected Features**				**SHAP** **Selected Features**			
**Classifiers**	**Acc**	**P**	**R**	**F1**	**Acc**	**P**	**R**	**F1**	**Acc**	**P**	**R**	**F1**	**Acc**	**P**	**R**	**F1**
Random Forest	0.63	0.60	0.63	0.61	0.68	0.71	0.68	0.67	0.57	0.44	0.57	0.50	0.42	0.34	0.42	0.38
Logistic Regression	0.63	0.67	0.63	0.64	0.57	0.61	0.57	0.57	0.68	0.70	0.68	0.68	0.42	0.41	0.42	0.41
SVM	0.57	0.55	0.57	0.56	0.63	0.63	0.63	0.63	0.52	0.52	0.52	0.52	0.36	0.38	0.36	0.37

**Key:** Acc: Accuracy, P: Precision, R: Recall, and F1: Weighted F1-Score

### 3.2 Optimization and hyper-parameterized tuning

In phase – 2, to produce a parsimonious and stable set of EEG features we applied a multi-stage supervised selection pipeline. First, we applied univariate ANOVA F-tests (SelectKBest, f_classif) to screen for features with significant between-class differences. In parallel, we computed MI scores to capture nonlinear single-feature associations with class labels. Next, we trained a RF classifier and extracted model-based feature importances (and verified them with permutation importance to reduce impurity bias). Finally, we computed SHAP values for the (tuned) model and aggregated mean (|SHAP|) per feature as a model-centric importance score. We then selected features that ranked highly across multiple scorers — operationalized as the intersection/consensus of the top-k features from ANOVA, MI, RF and SHAP — producing a final compact set of 7 features (3 change-based (∆) features and 4 post-KA features). Feature number K and ranking thresholds were tuned using LOOCV initially and final model selection and performance reporting used stratified 5-fold cross-validation shown in Table 4 to obtain stable estimates of expected generalization as it gives more stable results and robust estimate since each split has balanced class representation.

**Table 4:** LOOCV and StratifiedKFold Comparison.

**Model**	**LOOCV Accuracy**	**LOOCV F1**	**5-Fold Accuracy**	**5-Fold F1**	**Interpretation**
ModelA (XGB)	≈ 0.58	≈ 0.54	≈ 0.60	≈ 0.58	Learns some separation but tends to underfit smaller classes.
ModelB (RF)	≈ 0.68	≈ 0.67	≈ 0.75	≈ 0.70	Very stable; good balance between bias & variance. Works best with small EEG data.
ModelC (SVM)	≈ 0.68	≈ 0.60	**≈ 0.80**	**≈ 0.74**	Strongest generalization — performs best on unseen folds, meaning it captures consistent EEG feature patterns.
ModelD (Log Reg)	≈ 0.68	≈ 0.68	≈ 0.70	≈ 0.64	No added benefit — overlapping errors across models reduce diversity gain.

SVM (Model C) gives the highest 5-fold mean accuracy (∼80%), meaning that it generalizes best across small EEG folds, and it’s likely learning the non-linear separations between seizure levels (Low / Normal / High). Random Forest (Model B) remains the most robust backup model, slightly lower accuracy but more interpretable (feature-based).

### 3.3 Final model selection

**Fig. 4:**
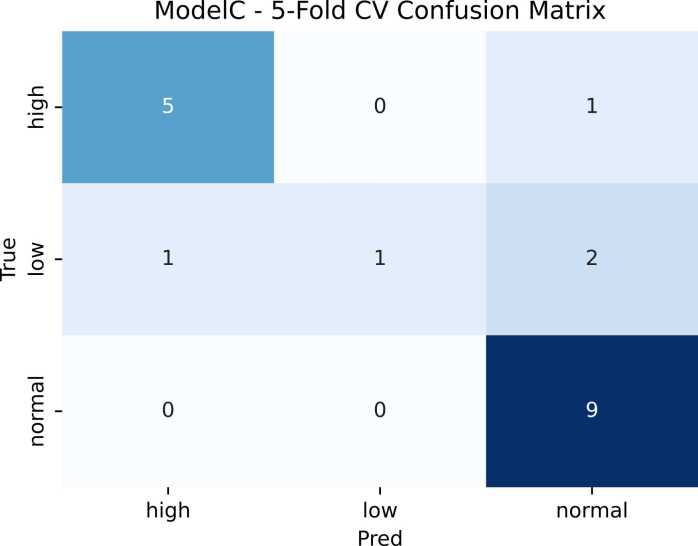
The 5-fold cross-validation confusion matrix demonstrates the model’s ability to differentiate EEG recordings across severity levels. Normal EEGs were classified with the highest recall (≈1.00), indicating that baseline neural dynamics were effectively captured. In contrast, Low-severity EEGs presented the greatest classification challenge, frequently confused with both High and Normal categories due to overlapping spectral characteristics in the delta–theta range. High-severity EEGs were largely identified correctly, although a small proportion were misclassified as Normal, likely reflecting residual high-frequency post-KA activity. The findings suggest strong discriminative power for Normal states and partial overlap between Low and High severity patterns, limiting the weighted F1-score to approximately 0.74.

SVM is considered the best and final model for predicting the severity level shown in Table 5 and confusion matrix shown in Fig. 4 which demonstrates how well a model’s predictions match the true class labels by reporting correct and incorrect classifications for each class (including false positives and false negatives).

**Table 5:** Best Model for severity classification.

**Class / Metric**	**Model C: SVC** **StratifiedKFold (5) CV**			
	Precision	Recall	F1-Score	Support
High	0.81	0.84	0.83	6
Low	1.00	0.25	0.40	4
Normal	0.75	1.00	0.86	9
Accuracy			0.79	19
Macro Avg.	0.86	0.69	0.70	19
Weighted Avg.	0.83	0.79	0.75	19

### 3.4 Most predictive EEG biomarkers

Feature importance analysis identified a consistent set of EEG biomarkers that were most informative for distinguishing severity levels within the training cohort of 19 mice. Notably, ∆_ch1_hjorth_complexity_mean and post_ch1_hjorth_mobility_mean reflected alterations in signal complexity and frequency variability, capturing increased neural irregularity and cortical excitability following KA administration. Spectral features such as ∆_ch2_rel_theta_mean and post_ch2_rel_alpha_mean revealed shifts in power distribution toward elevated theta and reduced alpha activity, patterns commonly associated with disrupted cortical synchronization and early epileptiform dynamics.

Higher-order statistical measures, including ∆_ch2_skew_mean and post_ch2_skew_mean, quantified waveform asymmetry, indicating non-linear distortions characteristic of pathological discharges. Additionally, post_ch1_zcr_mean highlighted increased temporal irregularity and oscillatory activity during seizure onset.

**Fig. 5:**
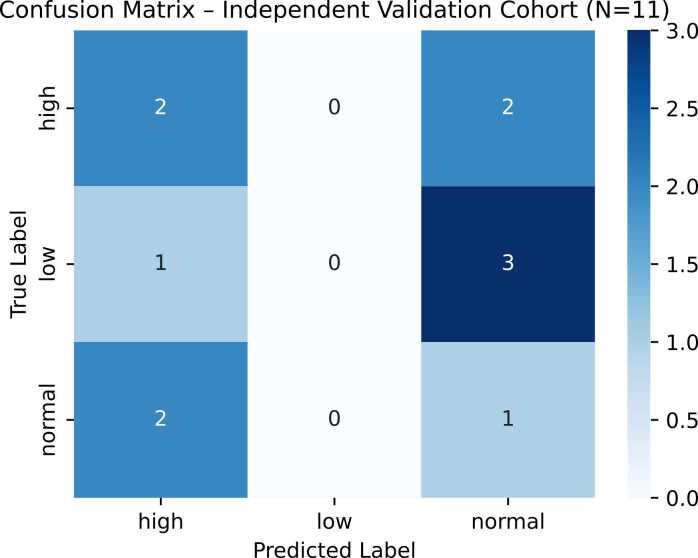
Confusion matrix illustrating classification performance on the independent validation cohort (N = 11 mice). The matrix highlights class-specific misclassification patterns, with reduced generalization performance compared to training data, reflecting biological and temporal variability across cohorts.

In addition to cross-validation within the training cohort, model generalizability was assessed using an independent validation dataset comprising EEG recordings from an additional cohort of (N = 11) mice collected separately from the primary training set (N = 19). These validation EEGs were not used at any stage of model training, feature selection, or hyperparameter optimization, and therefore represent a fully unseen test cohort. Overall classification accuracy was reduced in this independent validation cohort, with correct classification achieved in 3 out of 11 mice. Performance across severity classes is summarized in Fig. 5, which presents the confusion matrix for the unseen dataset.

This figure highlights class-specific misclassification patterns and illustrates the challenges of generalization when applied to a temporally and biologically independent cohort. This outcome highlights the substantial inter-animal variability and temporal heterogeneity inherent in post-status epilepticus EEG recordings. Importantly, despite reduced predictive performance at the cohort level, the same subset of EEG biomarkers identified during training consistently exhibited strong discriminative relevance, supporting their robustness as candidate prognostic indicators of spontaneous seizure burden severity. These findings emphasize that while classification performance may be limited under strict external validation, biologically meaningful EEG features can still be identified that reflect early pathophysiological processes driving epileptogenesis.

Following the initial feature screening, Phase-2 focused on identifying the most stable and biologically relevant EEG biomarkers by computing the intersection of four complementary feature selection techniques: ANOVA, Mutual Information, Random Forest importance, and SHAP values. This conservative strategy resulted in a final subset of seven EEG features that were consistently identified as highly informative across all methods.

**Fig. 6:**
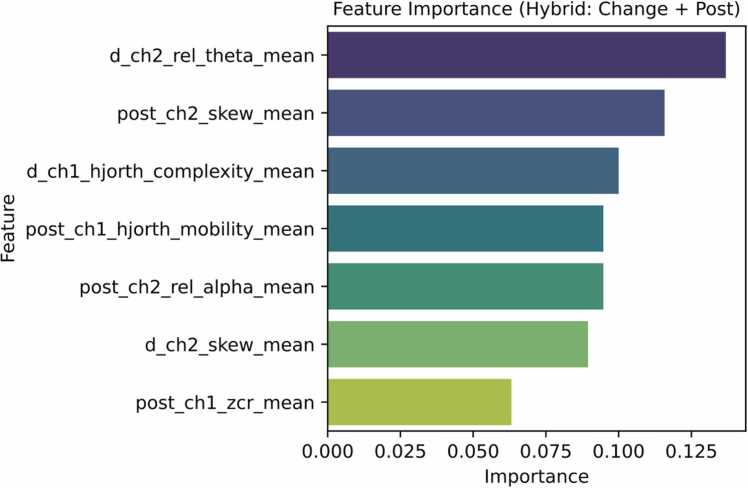
Importance of the final intersection-based EEG biomarkers. This figure shows the relative importance scores of the seven EEG features selected through the intersection of ANOVA, Mutual Information (MI), Random Forest (RF), and SHAP-based feature selection methods in Phase-2 of the analysis. The x-axis represents normalized feature importance values, while the y-axis lists the final set of consensus biomarkers. These features consistently ranked highly across multiple selection techniques, indicating robust and stable contributions to spontaneous seizure burden severity classification.

Fig. 6 illustrates the relative importance of these seven consensus features, highlighting their contribution to severity discrimination. The selected biomarkers capture complementary temporal, spectral, and non-linear signal characteristics, including changes in signal complexity, spectral power redistribution, and waveform asymmetry following KA administration. The convergence of multiple feature selection approaches on this reduced feature set supports their robustness and suggests that these biomarkers represent core electrophysiological signatures underlying early epileptogenic processes.

**Fig. 7:**
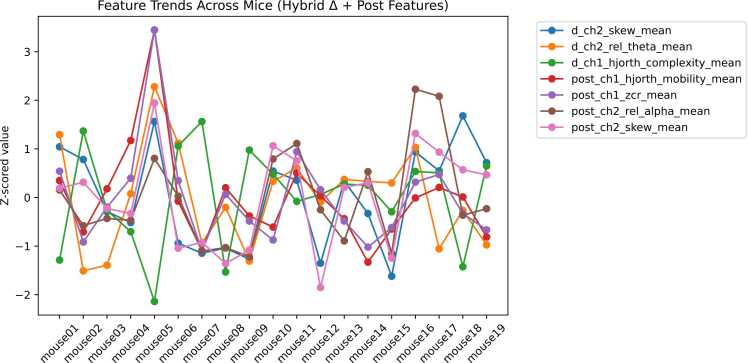
Reveals the Z-score trends across mice that indicates how many standard deviations a data point is from the mean of a dataset. They are used to standardize the values from different distributions, allowing comparisons, or to determine how far a value is from the average.

To further examine inter-animal variability, Z-score normalization was applied across mice to standardize feature values relative to the cohort mean and standard deviation. This transformation enables direct comparison of measurements recorded on different scales and highlights animals that deviate markedly from the group average. As shown in Fig. 7, the resulting Z-score profiles reveal distinct between-mouse trends, with positive and negative deviations indicating values above or below the cohort mean, respectively.

**Fig. 8:**
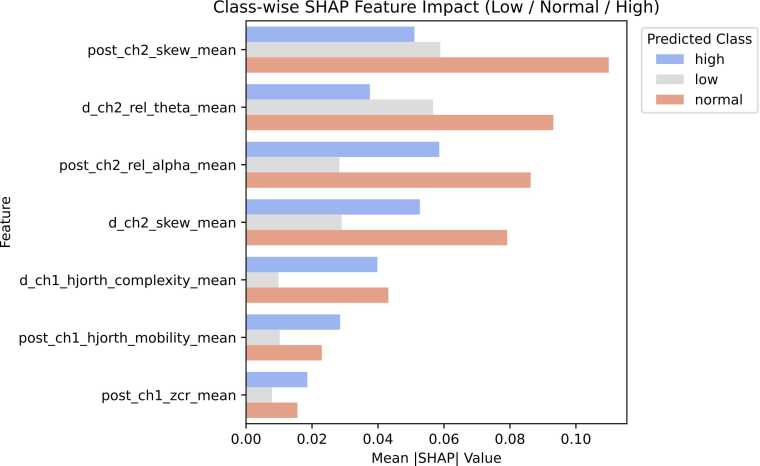
The SHAP plot shows the class-wise contributions from the final seven features. Each colour corresponds to a severity class (e.g., Low / Normal / High). Each bar shows how strongly a given feature contributes to predicting that class. For instance: For d_ch2_rel_theta_mean: The Red (Normal) bar is tallest means this feature contributes most strongly to Normal-severity predictions. Gray (Low) bar is moderate meaning some effect in low-severity mice, but weaker and Blue (High) bar is smallest that shows feature contributes the least to High-severity classification.

To improve interpretability of the multiclass classifier, SHAP analysis was used to quantify the class-specific contribution of the final seven EEG biomarkers to model predictions. This approach identifies how strongly each feature supports classification into the Low, Normal, or High seizure-burden groups. As illustrated in Fig. 8, several biomarkers showed differential importance across classes. For example, d_ch2_rel_theta_mean contributed most strongly to the Normal class, while showing weaker influence for Low and minimal contribution for High predictions. These findings indicate that individual EEG features carry distinct class-dependent predictive information rather than contributing uniformly across severity groups.

**Fig. 9:**
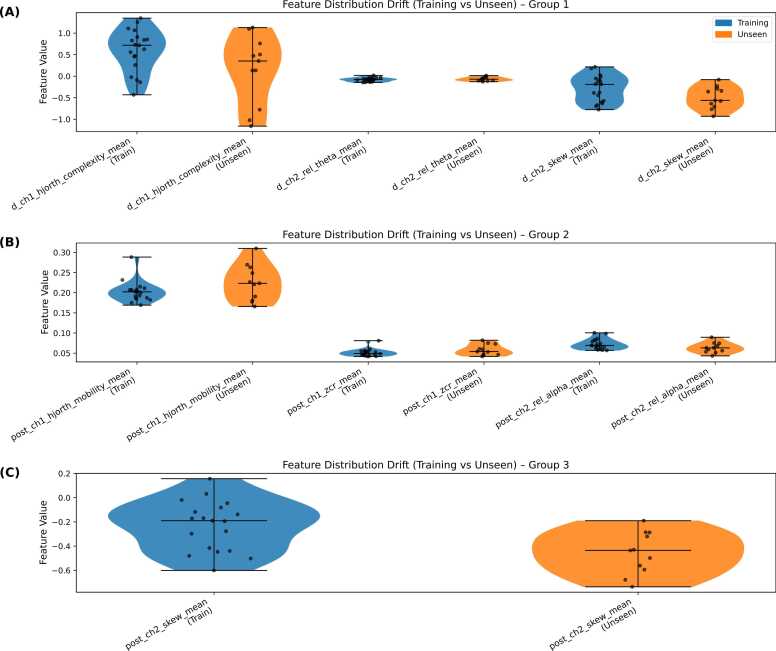
Violin plots illustrate the distribution of the seven most predictive EEG biomarkers (A-C) across the training cohort (Blue, N = 19) and an independent validation cohort (Orange, N = 11). Each violin represents the full distribution of feature values, with internal quartile markers indicating median and interquartile ranges. Individual data points are overlaid to illustrate sample-level variability and distributional differences between cohorts.

To further investigate the generalization gap observed between training and validation performance, violin plots shown in Fig. 9 were used to compare the distributions of the final seven EEG biomarkers across cohorts. Unlike summary statistics alone, these plots reveal the full density and variability of each feature. Several biomarkers demonstrated shifts in both median and distribution width in the independent validation cohort, suggesting differences in signal dynamics and spectral composition between datasets. This distributional divergence indicates that while these features were highly discriminative within the training cohort, their statistical properties were not fully preserved in unseen recordings, providing a plausible explanation for the reduced validation accuracy.

## 4 Discussion

The IAKA mouse model has been used for over 20 years to study the effects of seizures on the brain and identify novel therapies for epilepsy. However, a proportion of the mice in this model do not develop the typical rates of SRS. Since these mice may not be useful for drug screening, either because rates of SRS are too low to see effects of a drug or because of animal morbidity and risk of seizure-induced death, there is value in being able to identify these as soon as possible after the performance of IAKA. We previously found that by analysing just the first 40 min of EEG recordings after IAKA, during status epilepticus, we could predict with some confidence which mice would be normal or outliers (Edoho et al., 2025).

The present study demonstrates that short-duration (5-min) baseline and (∼40-min) post-KA EEG recordings, when combined with change-based (∆) and spectral–statistical features, can provide meaningful prognostic information about subsequent spontaneous seizure burden severity in the mouse IAKA model. Multiple supervised classifiers were evaluated under stringent cross-validation schemes. The strongest internal performance was achieved by the SVM model, supporting the hypothesis that early electrographic dynamics contain prognostic information relevant to subsequent spontaneous seizure burden. The findings support compliance with 3Rs principles and enable improved design, conduct and outcome tracking using this popular model of drug-resistant epilepsy.

Here we developed a model and performed feature selection hybrid multi-criterion selection approach (i.e., ANOVA, MI, RF, and SHAP). The intersection of top-ranked features from all four techniques was taken to ensure that only consistently informative features were retained across statistical, information-theoretic, and model-based criteria. This intersection strategy yielded a compact and interpretable subset of key EEG features, comprising both change-based (∆) and post-KA metrics. The classification of epilepsy phenotype remains an unknown task in the absence of appropriate discriminative markers (Sheikh et al., 2024). Our feature selection not only improves model performance and reduces the number of features by removing redundant features, but it also mines and clarifies the key aspects in differentiating between the low, normal, and high.

Importantly, the selected EEG features were able to distinguish patterns of brain activity recorded during the status epilepticus period that were associated with subsequent low, normal, or high seizure-rate phenotypes. These findings suggest that such features may serve as candidate biomarkers for differentiating mice at lower, intermediate, or higher risk of later seizure burden. This hybrid feature selection process achieved a strong balance between interpretability and predictive robustness. Among the evaluated models, the Support Vector Machine (Model C) achieved the highest predictive performance (Accuracy ≈ 0.79 and F1 ≈ 0.74), confirming that nonlinear feature boundaries more effectively capture the multivariate patterns differentiating Low, Normal, and High severity classes. The consistent superiority of the hybrid selected feature subset underscores the importance of nonlinear dependencies between EEG descriptors and seizure outcomes—dependencies that are often missed by univariate statistical methods.

At the biomarker level, the final feature set integrated both post-KA and change-based (∆) components, reflecting the dynamic neurophysiological shifts following excitotoxic injury. The dominance of Hjorth parameters and spectral power ratios in theta and alpha bands aligns with previous reports linking cortical desynchronization, high-frequency mobility, and altered thalamo-hippocampal coupling to epileptogenesis (Alarcon et al., 1995, Rizal et al., 2023). Specifically, increased Hjorth mobility and complexity in the ipsilateral hippocampal channel indicated heightened cortical excitability and irregular oscillatory dynamics, while elevated delta–theta activity and reduced alpha power in the contralateral channel reflected abnormal synchronization. These findings collectively suggest that both hemispheric interactions and temporal–spectral imbalance play central roles in defining the trajectory of severity development.

The confusion matrix analysis further revealed that the model achieved near-perfect recall for the Normal severity class, indicating a low false-negative rate for identifying animals that did not develop pathological seizure burden. In contrast, misclassifications predominantly occurred between the Low and High severity groups, reflecting overlapping electrophysiological signatures in delta and theta frequency bands during intermediate and severe epileptic states. From an experimental perspective, these errors were largely driven by false-positive predictions between Low and High severity categories rather than misclassification of Normal animals as pathological, which represents a more favourable outcome for severity screening studies. False negatives—where highly epileptic animals were predicted as lower severity—were comparatively less frequent, suggesting that the model retained sensitivity to pronounced pathological patterns despite reduced generalization performance. Such trade-offs between false-positive and false-negative rates are expected in early-stage severity stratification based on EEG, particularly when transitional neurophysiological states blur categorical boundaries. Similar trends have been reported in quantitative EEG studies of both rodent and human epilepsy, where intermediate phenotypes exhibit shared spectral characteristics that challenge strict class separation (Li et al., 2022, Segovia-Oropeza et al., 2025).

Our results extend work that primarily relied on seizure-period or long-term recordings by demonstrating that early post-KA EEG changes alone can yield meaningful severity prediction (Barker-Haliski et al., 2015). The hybrid feature selection framework, integrating ANOVA, MI, RF, and SHAP rankings, provided a robust and interpretable pathway to biomarker discovery—balancing statistical relevance with model-based evidence. Such interpretability is essential for translational EEG research, allowing correspondence between computational features and underlying neurophysiology. Future studies could expand on these findings by validating the current biomarkers in larger cohorts, incorporating additional modalities (e.g., LFP, behaviour metrics), and exploring deep learning architectures for end-to-end severity prediction while maintaining explainability (Roy et al., 2019).

The relatively high training accuracy and weighted F1-score obtained using SVM suggest that engineered EEG features, particularly those capturing deviations from baseline activity, effectively encode biologically meaningful alterations induced during status epilepticus. The convergence of four independent feature selection techniques (ANOVA, Mutual Information, Random Forest importance, and SHAP) further supports the stability and relevance of the selected feature subset. These findings align with prior studies reporting that early post-insult EEG abnormalities correlate with later spontaneous seizure burden and severity, reinforcing the role of early network dysfunction in disease progression (Bragin et al., 2004, Pitkänen et al., 2015).

Although the classifiers demonstrated strong performance within the training cohort, predictive accuracy decreased when evaluated on the independent validation dataset. This reduction likely reflects the substantial inter-animal variability and heterogeneous electrographic patterns observed following status epilepticus, as well as differences in seizure burden and recording duration across animals (Löscher, 2017, Temko et al., 2011). Importantly, the primary aim of this study was not to optimize classification accuracy per se, but to identify early EEG biomarkers that are informative of future epilepsy severity.

In this context, the consistency of the selected features across cross-validation and independent testing supports their biological relevance, even in the presence of reduced external predictive performance. Importantly, the presence of overlapping yet shifted distributions suggests that the selected biomarkers capture genuine neurophysiological signatures associated with spontaneous seizure burden severity, rather than spurious noise (Pitkänen et al., 2015). However, biological heterogeneity, variable seizure durations, and temporal differences in post-KA recordings likely influenced feature distributions in the validation cohort (Roy et al., 2019). These findings emphasize the importance of cohort-matched acquisition protocols and support the interpretation of the identified biomarkers as mechanistically informative, even in the absence of strong predictive generalization.

The observed divergence in feature distributions between training and unseen cohorts suggests a domain shift, providing a plausible explanation for the reduced external validation performance despite robust internal accuracy. Similar challenges in generalization have been reported in both experimental and clinical EEG-based epilepsy studies, underscoring the difficulty of translating complex neurophysiological signatures across cohorts (Truong et al., 2018).

Feature extraction relied on engineered statistical and spectral descriptors, which—while interpretable—may not capture complex nonlinear dynamics beyond second-order statistics (Craik et al., 2019). Future work could explore deep learning or temporal graph-based approaches to automatically learn discriminative patterns directly from raw EEG. Moreover, integrating multimodal data such as behavioural scoring, video-based seizure detection, or molecular biomarkers could further enhance predictive accuracy and translational relevance (Mirowski et al., 2008, Raghu and Schmidt, 2020).

Importantly, such generalization challenges have been widely reported in epilepsy prognostic modelling and do not necessarily invalidate the underlying biological signal. Instead, they highlight the sensitivity of ML models to cohort-specific characteristics when trained on small datasets, particularly in high-dimensional EEG feature spaces.

Despite these promising findings, several important considerations should be acknowledged. It is important to note that the present study focuses on early EEG dynamics during status epilepticus and their association with subsequent spontaneous seizure burden phenotypes within the experimental timeframe. While these findings provide insight into early electrographic markers of disease severity, they do not directly establish prediction of chronic epilepsy progression (Löscher, 2017, Pitkänen and Engel, 2014). Future studies incorporating longer-term longitudinal recordings will be necessary to determine the extent to which early EEG features can predict chronic epileptogenesis and enduring seizure susceptibility.

## 5 Limitations and future directions

This study is limited by the relatively small sample size and the absence of fine-grained seizure event annotations, which constrained the ability to model seizure frequency dynamics directly. Additionally, while time-normalized aggregation was employed to mitigate duration variability, residual distribution shifts likely persisted. Future work will focus on expanding the dataset, incorporating seizure-event-level information, and exploring domain adaptation strategies to improve cross-cohort generalization.

## 6 Conclusion

### 6.1 Overall significance

Taken together, these findings demonstrate that early EEG features recorded during status epilepticus carry meaningful prognostic information regarding spontaneous seizure burden severity in the IAKA model, while also illustrating the inherent challenges of translating internally validated models to independent datasets. This work provides a robust methodological framework for future studies aiming to bridge the gap between mechanistic EEG biomarkers and reliable prognostic tools in epilepsy research.

Finally, the present framework focuses on early post-KA recordings; extending this approach to longitudinal datasets could uncover temporal evolution of biomarkers and enable continuous severity monitoring. Such extensions would strengthen the clinical and ethical impact of early EEG-based prognosis, supporting more humane and data-efficient preclinical epilepsy research.

While reduced performance was observed on an independent unseen cohort, this outcome reflects the intrinsic heterogeneity of epileptic progression and the methodological challenges associated with small preclinical EEG datasets. Importantly, the model consistently identified animals that developed normal or low seizure burden, suggesting that early electrographic signatures may reliably capture non-progressive or resilient phenotypes. These findings support the notion that the nature of early network activity during status epilepticus plays a causal role in shaping long-term disease outcomes.

Overall, this work provides a principled and reproducible ML framework for spontaneous seizure burden severity prediction using early EEG recordings. The results underscore both the promise and the limitations of current prognostic modelling approaches and motivate future studies incorporating larger cohorts, standardized recording protocols, and longitudinal seizure characterization to enhance generalizability. Ultimately, such efforts may contribute to the development of early biomarkers for risk stratification and targeted therapeutic intervention in epilepsy.

## CRediT authorship contribution statement

**Omar Mamad:** Validation, Resources, Investigation, Data curation, Conceptualization. **Syed Muhammad Raza Abidi:** Writing – original draft, Visualization, Software, Methodology, Investigation, Formal analysis. **Gabriel-Miro Muntean:** Writing – review & editing, Supervision, Resources, Project administration, Methodology, Investigation, Funding acquisition. **David C. Henshall:** Writing – review & editing, Validation, Supervision, Project administration, Methodology, Investigation, Funding acquisition, Conceptualization. **Jordan Higgins:** Writing – review & editing, Validation, Methodology, Investigation, Formal analysis, Conceptualization.

## Ethical statement/standards

Mouse studies were carried out in accordance with the European Communities Council Directive (2010/63/EU). Procedures were approved by the Research Ethics Committee (REC 1587) of the RCSI University of Medicine and Health Sciences, under license from the Ireland Health Products Regulatory Authority (AE19127/P057).

## Declaration of Competing Interest

The authors declare that they have no known competing financial interests or personal relationships that could have appeared to influence the work reported in this paper.
